# Primary MALT Lymphoma of the Breast Treated with Definitive Radiation

**DOI:** 10.1155/2016/1831792

**Published:** 2016-05-10

**Authors:** Mohammad Hissourou III, Sayyad Yaseen Zia, Mahfood Alqatari, James Strauchen, Richard L. Bakst

**Affiliations:** ^1^Icahn School of Medicine at Mount Sinai, New York, NY 10029, USA; ^2^Department of Radiation Oncology, Icahn School of Medicine at Mount Sinai Hospital, New York, NY 10029, USA; ^3^Department of Pathology, Icahn School of Medicine at Mount Sinai, New York, NY 10029, USA

## Abstract

We are reporting a case of a 59-year-old woman, with a family history of breast cancer, who presented with extranodal marginal zone lymphoma (MALT) of the left breast. She received definitive radiation therapy and remains without evidence of disease. Here, we present a case and review the current literature to determine the optimal treatment of this rare presentation of MALT.

## 1. Introduction

Mucosa-associated lymphoid tissue (MALT) lymphomas are extranodal B cell lymphomas and a type of marginal zone lymphoma. The most common sites of MALT lymphomas are the stomach, spleen, and the eye/adnexa. MALT lymphomas of the breast are exceedingly rare. It has been hypothesized that the rarity of primary breast lymphomas, which account for just 0.4–0.5% of all breast malignancies and 1.7–2.2% of all extranodal lymphomas, is due to the scarcity of mucosa-associated lymphoid tissue in the breast [1, 2]. We report a case of primary breast MALT and we review the currently available literature on etiology, pathogenesis, diagnosis, prognosis, and treatment of this rare manifestation of a MALT.

## 2. Case Report

**Figure 1:**
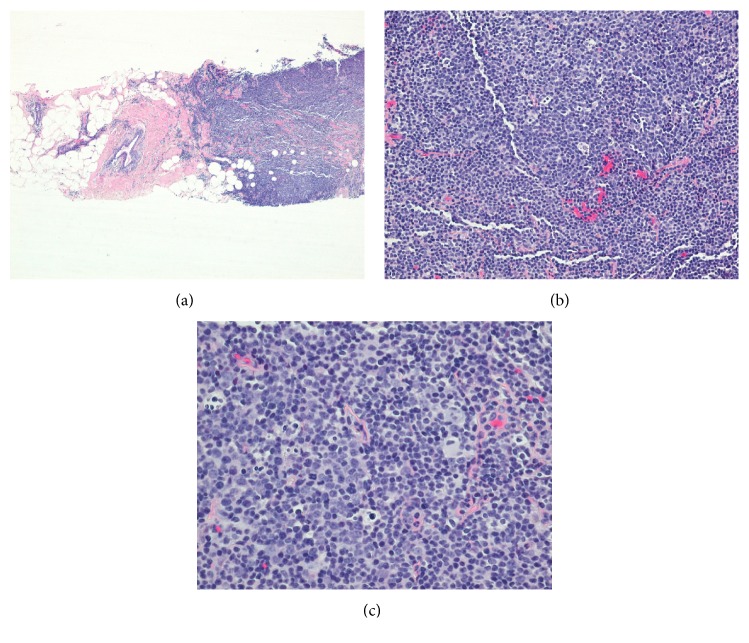
Low and high power images of H&E stain show infiltration of the breast tissue by lymphocytes of MALT lymphoma. These lymphocytes have abundant pale cytoplasm leading to monocytoid features.

The patient has been undergoing routine screening mammograms since the age of 40. On her annual screening mammogram, a 9 mm left breast mass was noted. She underwent a stereotactic core biopsy, which demonstrated an expansion of B cells found in irregularly shaped aggregates, which were associated with disrupted follicular dendritic cell meshworks. The B cells were CD20 positive, but negative for CD5, CD10, and BCL6; these findings were consistent with MALT of the breast (Figure 1).

**Figure 2:**
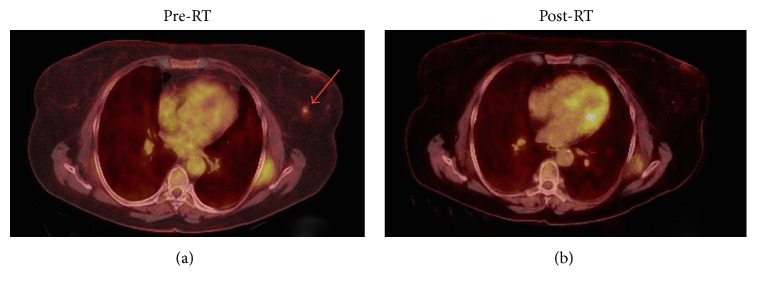
Pretreatment axial PET showing left breast mass with avidity in the left breast (a) and posttreatment axial PET showing complete response with no avidity present in the left breast (b).

The patient underwent staging workup, which included positron emission tomography (PET) imaging, computerized topography (CT) imaging, and a bone marrow biopsy. The PET scan showed mild uptake in an 8 mm left breast nodule (Figure 2(a)). Bone marrow biopsy was negative. The patient denied any B-symptoms and was staged as IAE MALT lymphoma of the breast.

**Figure 3:**
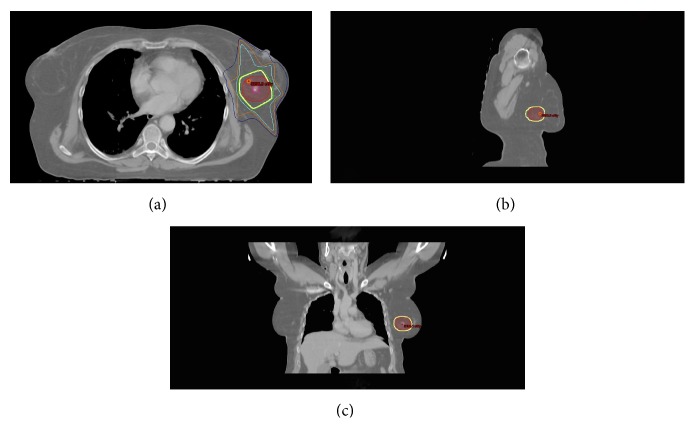
3D conformation radiation therapy planning of MALT breast lymphoma. Dose distribution in the axial (a), sagittal (b), and (c) coronal planes. The red area indicates the planning target volume (PTV) and the yellow line indicates the 30 Gy isodose line.

Treatment options were discussed, including lumpectomy and definitive radiation therapy. The patient elected definitive radiation therapy. The patient received definitive 3D conformational radiation therapy treatment to a total dose of 3000 cGy in 15 fractions (Figure 3). The treatment included the PET positive area with a small margin; the patient experienced no toxicity, including dermatitis, due to the minimally involved treatment area. At her three-month follow-up, she had no evidence of disease (Figure 2(b)).

## 3. Discussion

Non-Hodgkin lymphomas (NHL) are malignancies that originate in lymphoid tissue and arise from T cells, B cells, and natural killer (NK) cells, with MALTs representing only 5% of all NHL [3]. The etiology of NHL is poorly understood; however, the most significant risk factor for development of all NHL is immunodeficiency [4]. Interestingly, MALT lymphomas specifically have been associated with robust, prolonged inflammation in the case of* Helicobacter pylori* infection and with chronic immune system dysregulation in the case of Sjögren's syndrome and Hashimoto's thyroiditis [5–8].

The precise mechanism of the pathogenesis of breast MALT is not known; however, there is a hypothesis that sex hormones modulate immune function and the development of NHL. Nevertheless, findings concerning the specific role of estrogen in the development of NHL as a whole are mixed; some studies have found no effect, while others have found a protective or contributory effect [9–11]. Further research on the proliferative effects of estrogen in malignant conditions could be beneficial in the development of new treatments; there is some current evidence to support the proposition that estrogen receptor *β* may modulate the proliferative effects of estrogen receptor *α* [12]. Further research on the balance between proliferative (ER*α*) and antiproliferative (ER*β*) effects of estrogen receptors could lead to new NHL cancer treatments.

Patients with breast MALT may present with a unilateral palpable mass; however, most patients will be otherwise asymptomatic, including absence of classic B-symptoms [37, 38]. In a retrospective study of lymphomatous disease of the breast, the overwhelming majority of patients detected their disease by palpation of a breast mass rather than mammography [39]. Routine mammography may be useful in detecting MALTs. Once a diagnosis of MALT is established, the standard lymphoma workup including bone marrow analysis should be performed.

The prognosis of patients affected by breast MALT will depend, in part, on their clinical stage. Additional predictive factors include age, number of extranodal sites, performance status, and LDH levels. The treatment for primary breast lymphoma is not yet fully established. However, for localized MALT lymphomas, radiation therapy alone can be used as the definitive treatment. For localized breast MALT, local radiation therapy, such as involved field radiation therapy, and a moderate dose of 25–30 Gy are recommended [40, 41]. Treatment guidelines by the International Lymphoma Radiation Oncology Group report a recommendation for whole breast radiation therapy, noting that partial breast radiation can be considered in some cases; in our case, we opted for partial breast radiation given the small tumor size in relation to her breast [42]. Local radiation therapy can yield control rates and overall-survival rates over 90% [37]. For our patient, we used involved site radiation therapy to a total dose of 30 Gy with excellent results; the patient had no evidence of disease at her 3-month follow-up.

For patients with disseminated disease, treatment options may include a watch-and-wait approach, biological therapy, and/or chemotherapy. Since tumors are generally highly receptive to radiation therapy and chemotherapy, mastectomy need not be considered and wide excision is not necessary in the majority of cases [37].  Table 1 summarizes treatment management for all reported cases of breast MALT lymphoma to date. Of 32 patients, 5 were treated with definitive radiation therapy, 1 patient received palliative radiation therapy, 1 patient received no treatment, 6 patients received chemotherapy alone, and 19 patients received surgery (either surgery alone or surgery in addition to chemotherapy, radiation therapy, or both). Of those that received definitive RT therapy, none died from progressive disease. Of the 6 patients who received chemotherapy alone, 1 died of progressive disease. Of the 19 patients who received surgery, 2 died of progressive disease. These findings further support the recommendation for definitive radiation therapy as a reasonable treatment option for breast MALT.

For MALT lymphomas treated with radiation therapy as the sole treatment modality, relapse rarely occurs distantly [43]. If the patient's cancer was detected by mammography, they should continue to undergo annual screening. They should also be counseled on secondary malignancy and risk of coronary artery disease depending on the dose to the heart.

In summary, breast MALTs are generally an indolent disease with an asymptomatic presentation, including absence of B-symptoms. For localized disease, definitive radiation represents a reasonable treatment option with excellent response, local control, and minimal toxicity.

## Figures and Tables

**Table 1:** Characteristics of breast MALT lymphoma cases available in the English language with a minimum follow-up of 3 months and a reported disease status outcome.

Study	Age	Treatment	RT dose	Disease status	Follow-up (months)
Gupta et al., 2000 [13]	64	RT	44.2 Gy + R side boost up to 51 Gy	NED	64
Batstone et al., 2003 [14]	87	Palliative RT	(—)	Alive with disease	11
Ghetu et al., 2011 [15]	77	Lumpectomy; whole breast irradiation following breast relapse; rituximab following breast relapse; local RT following lacrimal gland relapse	(—)	NED	60
Tsang et al., 2001 [16]	74	RT for primary breast MALT and resection for Ca ampulla of Vater relapse	(—)	NED	55
Wright et al., 1996 [17]	55	RT	(—)	NED	168
Zobolas et al., 2002 [18]	68	Breast conservation surgery; bilateral axillary lymph node dissection; 6 cycles of CHOP and RT (RT given 6 months after surgery)	(—)	NED	10
Bailey et al., 1996 [19]	36	Excision, chemotherapy, and RT	(—)	NED	46
Rajendran et al., 2008 [20]	66	RT	4140 cGy in 23 fractions	NED	72
Matsuda et al., 2014 [21]	47	Mastectomy with sentinel node biopsy and radical axillary node dissection		NED	6
Arslan et al., 2012 [22]	69	CHOP protocol-8 cycles		NED	6
Michael et al., 2005 [23]	59	2 cycles of chlorambucil + CHOP		NED	24
Nassif and Ozdemirli, 2013 [24]	18	Excision		Alive with disease	4
Huber et al., 2002 [25]	32	8 cycles of cyclophosphamide, Oncovin, and prednisolone		NED	48
Julen et al., 2009 [26]	86	Patey surgery		NED	60
Kambouchner et al., 2003 [27]	37	No treatment		NED	42
Kim et al., 2015 [28]	55	Surgery		NED	9
Kuper-Hommel et al., 1999 [29]	65	3 cycles of CHOP		NED	10
Mattia et al., 1993 [30]	69	Excision		NED	9
Mattia et al., 1993 [30]	77	Excision		NED	48
Mattia et al., 1993 [30]	81	Excision		NED	10
Mattia et al., 1993 [30]	65	Excision		Death from progressive disease	25
Raderer et al., 2005 [31]	59	4 cycles of oxaliplatin		NED	20
Said et al., 2013 [32]	52	Cyclophosphamide, steroid, and CHOP		Death from progressive disease	12
Taeda et al., 2006 [33]	84	Mastectomy with axillary lymph node dissection and 4 cycles of rituximab		NED	18
Welsh et al., 2006 [34]	66	Lumpectomy and 30.6 Gy in 18 fractions		NED	36
Anavekar et al., 2008 [35]	56	Surgery, postoperative RT, and tamoxifen		NED	24
Kuper-Hommel et al., 2003 [36]	(—)	RT	(—)	Death from unrelated causes	59
Kuper-Hommel et al., 2003 [36]	(—)	Surgery		NED	134
Kuper-Hommel et al., 2003 [36]	(—)	Surgery and anthracycline-containing chemotherapy regimen		NED	74
Kuper-Hommel et al., 2003 [36]	(—)	Surgery and chemotherapy		NED	66
Kuper-Hommel et al., 2003 [36]	(—)	Surgery		NED	16
Kuper-Hommel et al., 2003 [36]	(—)	Surgery and anthracycline-containing chemotherapy regimen		Death from progressive disease	107

SOB: shortness of breath.

CXR: chest X-ray.

NED: no evidence of disease.
